# Physiological Stress in Koala Populations near the Arid Edge of Their Distribution

**DOI:** 10.1371/journal.pone.0079136

**Published:** 2013-11-12

**Authors:** Nicole Ashley Davies, Galina Gramotnev, Clive McAlpine, Leonie Seabrook, Greg Baxter, Daniel Lunney, Jonathan R. Rhodes, Adrian Bradley

**Affiliations:** 1 School of Biomedical Sciences, The University of Queensland, St Lucia, Queensland, Australia; 2 Landscape Ecology and Conservation Group, School of Geography, Planning and Environmental Management, The University of Queensland, St Lucia, Queensland, Australia; 3 Office of Environment and Heritage New South Wales, Hurstville, New South Wales, Australia; 4 School of Veterinary and Life Sciences, Murdoch University, Perth, Western Australia, Australia; The University of Wollongong, Australia

## Abstract

Recent research has shown that the ecology of stress has hitherto been neglected, but it is in fact an important influence on the distribution and numbers of wild vertebrates. Environmental changes have the potential to cause physiological stress that can affect population dynamics. Detailed information on the influence of environmental variables on glucocorticoid levels (a measure of stress) at the trailing edge of a species’ distribution can highlight stressors that potentially threaten species and thereby help explain how environmental challenges, such as climate change, will affect the survival of these populations. Rainfall determines leaf moisture and/or nutritional content, which in turn impacts on cortisol concentrations. We show that higher faecal cortisol metabolite (FCM) levels in koala populations at the trailing arid edge of their range in southwestern Queensland are associated with lower rainfall levels (especially rainfall from the previous two months), indicating an increase in physiological stress when moisture levels are low. These results show that koalas at the semi-arid, inland edge of their geographic range, will fail to cope with increasing aridity from climate change. The results demonstrate the importance of integrating physiological assessments into ecological studies to identify stressors that have the potential to compromise the long-term survival of threatened species. This finding points to the need for research to link these stressors to demographic decline to ensure a more comprehensive understanding of species’ responses to climate change.

## Introduction

Stressors are pervasive and include environmental and ecological disturbances that have profound effects on the ecology and evolution of organisms [1]–[3]. The sources of stress include biotic factors (predation, competition, social dynamics), extremes in physical factors (temperature, salinity) and climatic factors (drought, storms) [3], [4]. Shifts in the environment can be labile (i.e. eventually subside, such as cold, storms, heat and drought) or permanent, such as global climate change, urbanization, habitat degradation, pollution, with permanent changes requiring more than just temporary assimilation and they can result in changes in range, adaptations or local extinctions [5]. Stressors can impact on both physical and biotic components of an organism’s environment and, depending on their pervasiveness, magnitude and frequency, can profoundly influence the fitness of individuals via costs to health, reproduction and survival [6]. Ultimately, stressors can have profound effects on population viability, species’ distribution and extinction risk [7]–[9]. Unfavourable conditions trigger physiological responses that result in hypothalamic-pituitary-adrenal (HPA) axis activation and glucocorticoid secretion (cortisol in marsupials) by the adrenal cortex [10]–[13]. These glucocorticoid hormones affect physiological and behavioural traits that regulate the responses of vertebrates to stressors, and potentially offer a metric for evaluating species risk to global change [6]. Thus, physiological investigations of wild animals are vital to identify specific conservation concerns and identify threat status. Depending on the type of sample collected, they can also offer a potentially non-invasive way to monitor the efficacy of management strategies [14].

In response to a stressor, the HPA axis is activated, resulting in the increased secretion of glucocorticoids (primarily cortisol and corticosterone) from the adrenal gland (stress response) [15]–[19]. Glucocorticoid receptors are distributed widely in the body, thus glucocorticoid hormones can initiate a wide range of responses affecting behaviour, reproduction, growth, the immune system, and metabolism and energy allocation [20]–[23]. The stress response seeks to reduce the negative effects of a stressor and may cause temporary suppression or interruption of an individuals normal life history stage (such as delays in reproduction, development, migration) to redirect resources into an ‘emergency’ state to facilitate immediate survival [4], [24]–[27]. When an emergency life-history stage is triggered, it allows an individual to temporarily move away from the source of disturbance (adjustment) or endure it while adopting energy-saving physiological strategies (adaptation) that allow it to cope [24]. As a consequence, glucocorticoid secretion resulting from acute stress (e.g. predator attack) improves the fitness of an organism by mobilizing energy during the stressful situation [22], [28]–[31]. However, a stress response involving excessive glucocorticoid secretion, often termed allostatic overload or chronic stress, arises when an individual’s HPA axis is challenged by excessive stressor exposure or pervasiveness resulting in fitness loss [32], [33]. Evidence from the biomedical literature shows that sustained, high levels of glucocorticoids can cause deleterious effects on the physiological health of an animal and lead to greater susceptibility to disease and reduced fecundity and survivorship [12], [13], [20], [34], [35]. Accordingly, variation in the release of glucocorticoids, as a measure of the stress response, is increasingly being used in ecological and conservation studies to infer the health of animals [23] and to enable us to understand how both natural (e.g. predators, conspecifics, weather) and imposed environmental challenges (e.g. climate change, relocation and habitat disturbance) impact on individuals and populations [36]–[40].

While the deleterious effects of chronic stress are theoretically well conceptualized from biomedical evidence, there are few empirical studies showing fitness loss or pathology and ensuing demographic consequences of allostatic overload in wild populations [6], [41]. Exceptions include a few studies on wild populations showing predation pressure causing indirect and negative glucocorticoid-mediated effects on individual fitness, resulting in decreased prey population growth [42]–[44]. Boonstra [41] contends that although chronic stress does occur in nature, the impacts of chronic stressors as laid out in the biomedical literature (i.e. pathology) do not occur in nature. Boonstra [41] argues that both demographic (the indirect effects on reproduction) and hormonal evidence indicate that although some wild populations may be chronically stressed by predators, there is no evidence of pathology or an HPA axis with an inability to respond. Boonstra [41] suggests that chronic stress is not the inevitable result of exposure to a persistent, severe stressor, but is part of the normal experience of many animals in nature and, although there are fitness costs, it is an evolved adaptive response to particular ecological and habitat pressures – i.e. trade-off reproduction for survival but not to such an extent that survival itself is at risk. Different views of the impacts of chronic stressors, as laid out in the biomedical literature relating to pathology and the impacts within natural populations, must be taken into consideration when assessing glucocorticoid concentrations from wild populations.

Non-invasive methods for measuring glucocorticoid metabolites in faeces and hair have become a widely accepted tool for gaining important information about an animal’s endocrine status [12], [45]–[48]. Faecal glucocorticoid metabolite analysis to assess physiological stress has been validated using different methods and employed in studies of several marsupial species including the honey possum (*Tarsipes rostratus*) [49], tammar wallaby (*Macropus eugenii*) [50], Gilbert’s potoroo (*Potorous gilbertii*) [51], the southern hairy-nosed wombat (*Lasiorhinus latifrons*) [52], the numbat (*Myrmecobius fasciatus*) [53] and the koala (*Phascolarctos cinereus*) [54], [55].

Unlike measurements of plasma and serum hormone concentrations that measure the actual hormone, non-invasive methods measure various metabolic end products of the hormone after it has been cleared from the circulation and extensively modified by bacteria in the gut [47], [56]–[59]. A specific hormone is typically broken down into various metabolites during this process [59], and it is these glucocorticoid metabolites that are measured in faeces (faecal glucocorticoid metabolites) and reflect the free (unbound) glucocorticoid fraction of total glucocorticoids [60], [61]. However, there are a number of caveats relating to sex, diet, metabolic rate and individual differences in hormone metabolite formation that are the source of complications that need to be considered for a meaningful interpretation of hormone metabolite data (for a review see [62], [63]). Despite these caveats, it has been demonstrated that faecal glucocorticoid metabolite levels can reflect both baseline glucocorticoid levels and an animal’s stress response [61], making this non-invasive technique a valuable alternative to invasive blood sampling in field studies [63]. Faecal glucocorticoid measures also provide an integrated measure of fluctuating blood concentrations in the preceding few days, thus providing a stable measure of an individual’s average hormone secretion [64]–[66]. Furthermore, faecal glucocorticoid measures can be obtained easily without handling or observing the animal, and thereby not elevating stress levels [12], [65], [67], [68].

As a consequence of a changing climate, distributional changes are occurring at the boundaries of geographic ranges (i.e. at the range limit) of many species, including boundary expansions at the leading edge of a range and contraction at the trailing edge [69]. Populations, in particular of folivores, at the trailing edge of their range, are likely to be most vulnerable to climate change through physiological stress associated with the decline in the nutrient richness of their food source [70], [71]. Trailing edge populations can be critical to the long-term survival of species because they may contain individuals that can adapt to changing climatic conditions [72]–[74].

The koala (*Phascolarctos cinereus*) is an arboreal, marsupial folivore, which feeds almost exclusively on a limited variety of *Eucalyptus*, *Corymbia* and *Angophora* species. It is widely distributed, with its range extending across 30 bioregions from tropical Queensland to temperate Victoria and South Australia. The koala was listed in May 2012 under the Commonwealth’s *Environmental Protection and Biodiversity Conservation Act 1999* as threatened in Queensland, the Australian Capital Territory and New South Wales. Previous research in southwestern Queensland, near the arid edge of their range, has determined that its distribution is directly affected by climatic factors (high temperature and low rainfall), which supports the view that these western koalas are near their physiological limits [75], [76]. Therefore, western koalas can be expected to cope poorly with climate change.

Significant effects of season or weather conditions (e.g. temperature, humidity, food and water availability) on glucocorticoid concentrations have been shown for several mammalian species, with most studies reporting higher levels during harsher conditions in winter, extreme heat, or during the dry season [66], [77]–[82]. Furthermore, a number of studies have attributed population declines to the effects of chronic stress in free-living mammals [36], [83], [84]. Additionally, a study on a long-lived seabird, the black-legged kittiwake, found that initial increases in allostatic load can evoke increased foraging behaviour and consumption, triggered by increasing glucocorticoid levels, which resulted in the accumulation of energy stores [85]. However, the metabolic effects of increased glucocorticoid levels experienced under high allostatic loads lead to a reduction of energy stores (indicating high physiological stress), with observations of decreased fitness (increased mortality, decreased reproduction) with high glucocorticoid levels in this species [85]. Throughout the koala’s distribution, droughts and heatwaves have been responsible for population crashes and local extinctions [71], [86]–[88]. In southwestern Queensland, there was an 80% decline in koala numbers between 1995 and 2009, with drought being the main contributor to the decline [88]. Climate change predictions for this region indicate that the intensity and frequency of droughts and heatwaves will increase, and moisture availability will decrease [89]–[91]. Therefore measuring glucocorticoid concentrations of western koala populations will help us understand how climatic variables impact these populations, and allow us to anticipate where problems will arise and thereby highlight specific conservation concerns [14].

The aim of this study was to investigate the effect of climate variables on the physiological stress of koala populations of southwestern Queensland to predict the potential response of these populations to future climate change. Koalas in this region are at the western limits of their geographic range and form a trailing-edge population [88]. We used non-invasive techniques to measure glucocorticoid levels. Mixed effects modeling was used to relate the current physiological conditions of koalas to current climate and environmental conditions. These relationships were then used to estimate potential physiological conditions based on predicted future climates.

## Materials and Methods

The approved protocol for this project was approved by the Animal Ethics Committee at The University of Queensland (GPA/603/08/ARC) and the Environmental Protection Agency (now the Department of Environment and Resource Management) (Scientific Purposes Permit WISP05343008).

### Study Area

**Figure 1:**
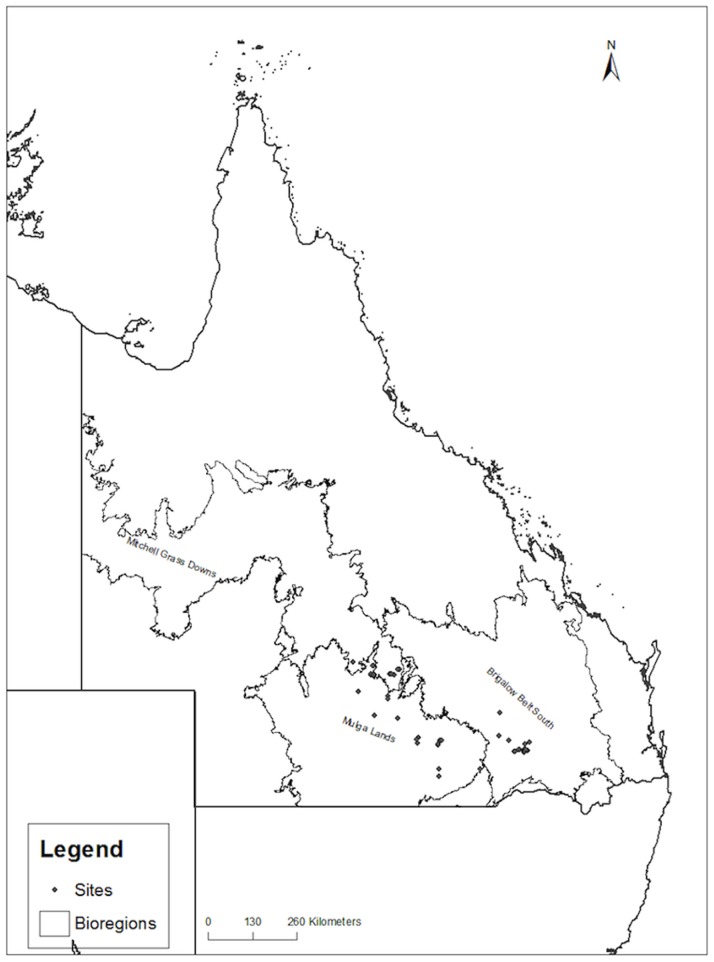
Study area within the Mulga Lands, Mitchell Grass Downs and Brigalow Belt South bioregions, Queensland, Australia. ‘Sites’ denote locations where fresh faecal pellets were collected.

This study was conducted in semi-arid, southwestern Queensland, comprising portions of the Mulga Lands bioregion, the Mitchell Grass Downs bioregion and the Brigalow Belt South bioregion (Figure 1). Annual average rainfall ranges from 750 mm in the east declining to 250 mm in the west. Rain falls mainly in summer and is highly variable [92]. The hottest month is January with mean monthly maximum and minimum temperatures of approximately 35°C and 19°C respectively. The coolest month is July with mean maximum and minimum temperatures of approximately 19°C and 4°C respectively [92].

The Mulga Lands bioregion is dominated by flat to undulating plains and low ranges supporting *Acacia aneura* (mulga) shrubland and low woodlands [93]. The woodlands that dominate waterways and associated floodplains are predominantly comprised of *Acacia* spp., *Eucalyptus populnea* (poplar box), *E. camaldulensis* (river red gum), *E. coolabah* (coolabah) and *E. orchophloia* (yapunyah) [93].

The western portion of the Brigalow Belt South bioregion is predominantly comprised of *Acacia harpophylla* (brigalow), *Casuarina cristata* (belah) and *E. populnea* open-forest woodland [93]. Riparian vegetation is dominated by *E. camaldulensis*, *E. coolabah* and *E. largiflorens* (black box) [94].

The Mitchell Grass Downs bioregion is dominated by treeless plains of *Astrebla spp.* (Mitchell grass) with some occasional ridges, rivers and gorges. Patches of low open woodland of *E. coolabah* occur across the region in association with low-lying plains and drainage lines [94].

### Faecal Pellet Collection

Searches were conducted to collect fresh koala faecal pellets for cortisol analysis in 29 sites across the three bioregions (Mulga Lands 13 sites; Brigalow Belt South 11 sites; Mitchell Grass Downs 5 sites) (Figure 1) between August 2009 and June 2011. Morning (07∶00–10∶00 hours), afternoon (16∶00–18∶00 hours) and night (spotlighting, 19∶00–04∶00 hours) searches were conducted in a systematic manner along transects (1 km by 80 m) within riparian habitats. Previous research had identified riparian habitats as the primary resource used by koalas in southwestern Queensland [88], [95]. If a koala was sighted along a 1 km transect, then an extra 1 km was surveyed to locate additional koalas. Once a koala was located, sex was recorded and faecal pellets were collected by searching the entire area beneath the canopy of the tree in which the koala was located. Fresh, intact pellets were placed in clean containers using forceps and stored in the field in a liquid nitrogen dry shipper (MCE SC 20/12 V Vapour Shipper, In Vitro Technologies Pty. Ltd) for transport to the laboratory. The use of faecal cortisol analysis in the assessment of the activity of the HPA axis in freshly collected koala faecal pellets has previously been validated using the methods described below [54]. That research showed an excretion time lag with faecal cortisol metabolite concentrations corresponding to the release of glucocorticoids from the adrenal cortex at least 36 hours prior to deposition [54]. Other studies have also successfully assayed cortisol metabolites in koala faeces (with an ACTH challenge), with time lags of 24 and 48 hours in females and males respectively [55].

### Extraction and Analysis

Faecal pellets from individual koalas were oven dried at 70°C for 10 hrs. The dried samples were crushed between two 10 mm PVC plates, passed through a 1 mm sieve to remove leaf particles, and collected into 5 ml flat bottom polypropylene screw capped vials. For each sample, 200 mg (±1 mg accuracy) of dry faecal powder was weighed into a 16×100 mm glass test tube. Borate buffer 2 ml (pH 6.5 0.1 M) was added to the dry powder, vortex mixed and then 50 µl of β glucuronidase (β-D-Glucuronoside glucuronosohydrolase, EC 3.2.1.31, Sigma Chemical Co. USA) containing approximately 4,000 units was added to each test tube to cleave glucuronide and sulfatase polar groups. Test tubes were then incubated for 4 hrs at 37°C on an orbital mixer. Redistilled diethyl ether 3 ml was added to each test tube and vortexed for 2 minutes, then the test tubes were allowed to stand for 2 minutes. The lower aqueous phase was frozen by briefly dipping the test tubes into liquid nitrogen and the supernatant ether containing the partitioned steroid was decanted into 12×75 mm glass test tubes and evaporated to dryness at 40°C in a hot block evaporator in a fume hood. The residue containing extracted steroid was re-dissolved in 200 µl of diluted zero cortisol calibration solution (Demeditec Saliva Free Cortisol Kit - diluted 1∶10) and placed on an orbital mixer at 37°C for 60 minutes, followed by short, high-speed vortex (20 seconds). 100 µl of test samples, standards and controls were then pipetted into 96 well plates of Saliva Free Cortisol Kits (Demeditec) and run as per kit directions. The efficiency of the extraction process was progressively tracked by addition of 30,000 dpm ^3^H-cortisol (1,2,6,7 ^3^H cortisol 160 Ci/mmol Perkin-Elmer Life Sciences) and the final cortisol assay concentration was corrected for this efficiency. This assay allowed us to measure cortisol after first cleaving polar bonds in the major known metabolites (faecal cortisol metabolite: FCM), because it has been reported that there are no native, unmetabolized glucocorticoids present in faeces [60].

The specificity of the assay was: cortisol 100%, corticosterone 0.38%, cortisone 1.85%, 11-deoxycortisol 0.88%, prednisolone 9.89%, with all other steroids of similar structure <0.20%. Inter-assay variation was 5.4% CV, while intra-assay variation was 5.5% CV. Analytical sensitivity of the assay is 0.014 ng/ml or 4.2 pg per loaded sample. Serial dilutions of glucuronidase-treated koala faecal extracts run against Demeditec assay kit calibrator standards gave a satisfactory degree of parallelism for the assay. Assay data were analysed employing a four parameter logistic fit using MyAssays Analysis Software Solutions (www.myassays.com).

### Explanatory Variables

The explanatory variables included sex, climate data, soil characteristics and location (Table 1). Climate data from the Bureau of Meteorology (1990–2011) were examined for stations closest to each site (within the same bioclimatic region and <30 km apart). Minimum and maximum average temperatures were considered for each sample corresponding to the day prior to sample collection to take into account the excretion time lag [54] (Table 1). Monthly rainfall was calculated because of time lags between rainfall and the response by vegetation [96]–[98]. Soil properties (e.g. total nitrogen and phosphorus) at each site were derived from geology mapping of Queensland (The Queensland Combined Soils dataset) at a scale of 1∶250,000 to 1∶200,000,000 (Table 1). Each site was also categorized by bioregion, catchment, basin, drought or post-flood event and season (Table 1).

**Table 1:** Explanatory variables used to examine variation in faecal cortisol concentrations.

Variable	Units	Full description
Sex	Categorical	Sex of each koala from which faecal samples were collected.
Total Nitrogen	Kjeldahl	Soil total nitrogen content at each site - derived from geology mapping of Queensland (The Queensland combined soils dataset).
Phosphorous	Kjeldahl	Soil phosphorous content at each site - derived from geology mapping of Queensland (The Queensland combined soils dataset)
Annual rainfall	Millilitres	Average annual rainfall (1990–2011) for each site.
Rainfall 1–12 months	Millilitres	Total rainfall with monthly resolution at each site for 1–12 months prior to each faecal sample collected.
Temperature	Degrees Celsius	Average minimum and maximum temperatures at each site the day prior to sample collection.
Bioregion	Categorical	Bioregion (Mulga Lands, Mitchell Grass Downs, Brigalow Belt South)
Catchment	Categorical	Catchment (Mungallala, Warrego, Condamine, Moonie)
Basin	Categorical	Basin (Warrego, Moonie, Balonne-Condamine)
Drought or post-flood	Categorical	Categorized based on whether each sample was collected during the drought (2009) or post-flood event (2010 onwards).
Season	Categorical	Categorized based on whether each sample was collected during the koala breeding season (September to March) or non-breeding season (April–August).

### Statistical Analysis

Statistical analyses were conducted using the Stata statistical software package [99] to determine the dependencies between the following variables: FCM concentration (ng/g) (dependent variable) and rainfall, temperature, soil nitrogen, soil phosphorus, sex, time of the year (month), and season (breeding/non-breeding) (independent variables – Table 1). As was demonstrated from the Shapiro-Wilk test for normality of data, the FCM (dependent) variable was not distributed normally (*p*<0.001). Therefore, the FCM variable was transformed logarithmically to achieve a normal distribution of the dependent variable. The explanatory variables were all standardized to have a zero mean and a standard deviation of one to allow comparison between the model parameter estimates.

Data were collected at 29 sites across the three bioregions. Therefore, we assumed a hierarchically structured population with random sampling of the regions, sites and subjects within the sites. Multilevel (three-level) mixed-effects linear regressions with random intercepts were used to assess the effect of each explanatory variable on koala FCM levels. The three levels within the considered model were associated with: (1) individual koala; (2) the site of data collection; (3) the region where the site was located. The random effects model determined the dependency of FCM levels on a particular rainfall variable (e.g., two month rainfall) for each site and/or region. Thus the random effects within the model are random intercepts and/or slopes for the respective regression lines of FCM levels versus a rainfall variable for each region and/or site. Though the model does not directly give the values of the random effects (random intercepts and/or slopes), their best linear unbiased predictions, also known as empirical Bayes predictions, can be calculated for both region and site levels [100].

## Results

**Figure 2:**
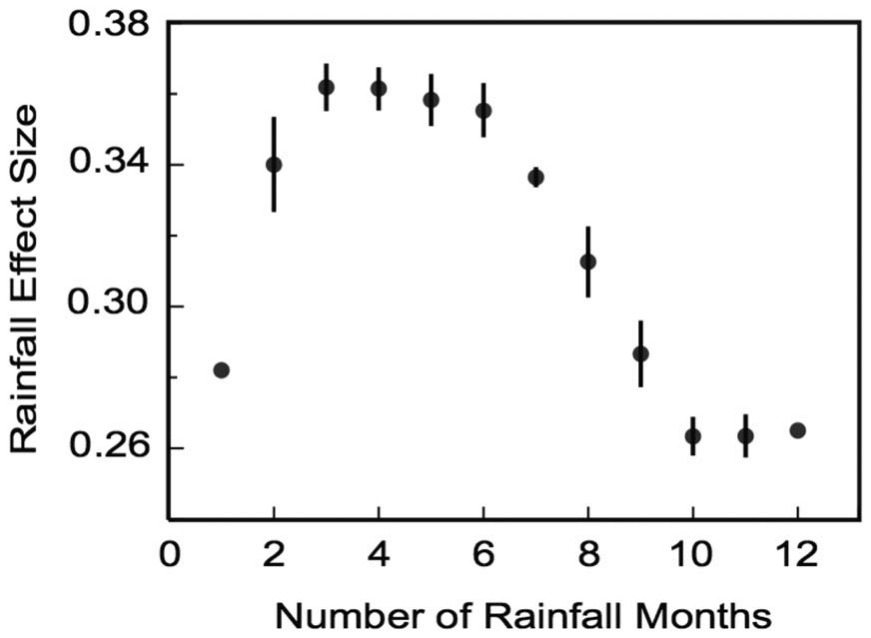
The moving average dependence of the effect size (standardized coefficients in Table 2) for the rainfall variable versus number of months with rain prior to data collection. The moving interval over which averaging is undertaken contains three consecutive values of the coefficients from Table 2. The first and the last points are added to this dependence directly from Table 2 (because these edge points are not included into the moving average dependence with the three-point moving interval). Error bars calculated as the errors of the mean within the moving interval are shown for each of the moving average points. Note, this figure shows the magnitude of the effect size, and the actual effect of rainfall on cortisol levels is negative.

All explanatory variables (Table 1), other than the covariates related to rainfall, were omitted from the considered model, as they had no significant impact on the levels of FCM (*p>*0.1). FCM concentration was assessed against rainfall for 1 to 12 months prior to sample collection (Table 2). The moving average dependence of the magnitudes of the standardized coefficients (Table 2) on the number of rainfall months is illustrated by Figure 2. Changes in FCM levels have a typical time lag of about 2–6 months after rain. This follows from the maximum of the curve in Figure 2, between ∼2 and 6 months. Further, the points corresponding to 4, 5 and 6 months in Figure 2 show the effect size for the 2 months rainfall. This means that the effect size for months 4, 5 and 6 is enhanced by the effect size for the 2 months rain. Thus, the strongest impact on FCM levels comes from the rainfall between ∼2–3 months prior to the collection time.

**Table 2:** Statistical effects of rainfall for one to 12 months prior to sample collection on faecal cortisol concentration (Coefficients from standardized models (effect sizes)).

Independent Variable	Coefficient	Standard error	P
Rainfall 1 month prior	−0.28	0.11	0.02
Rainfall 2 months prior	−0.391	0.095	<0.001
Rainfall 3 months prior	−0.347	0.096	<0.001
Rainfall 4 months prior	−0.348	0.097	<0.001
Rainfall 5 months prior	−0.39	0.11	<0.001
Rainfall 6 months prior	−0.34	0.10	0.001
Rainfall 7 months prior	−0.34	0.11	0.002
Rainfall 8 months prior	−0.34	0.11	0.001
Rainfall 9 months prior	−0.27	0.11	0.013
Rainfall 10 months prior	−0.26	0.10	0.013
Rainfall 11 months prior	−0.26	0.10	0.01
Rainfall 12 months prior	−0.27	0.10	0.01

Therefore, the model for the impact of rainfall on levels of FCM included two independent variables: rainfall for two months prior to the time of collection of the faecal pellets and rainfall for four months (3 to 6 months) prior to the time of collection. The variable of rainfall for months 3 to 6 prior to time of collection was included because it takes into account the decaying (but still extant) impact of rainfall during earlier months before the two-month period to the time of collection and its statistical significance (with the standardized coefficient being –0.25 and *p* = 0.012) identifies the long period over which the effect is evident (Table 3).

**Table 3:** Statistical effects of rainfall 2 months prior and rainfall 3, 4, 5 and 6 months prior (combined) on faecal cortisol concentration (standardized).

Independent Variable	Coefficient	Z	P
Rainfall 2 months prior	−0.2647747	−4.11	0.000
Combined rainfall for months3, 4, 5 & 6 prior	−0.1715594	−2.51	0.012

We did not find significant, non-linear quadratic terms in the dependence of FCM levels on two-month rainfall. Further, since comparing the fit of the random intercept model to that of the multiple regression model, using the likelihood ratio test yielded *p* = 0.01, we can thus reject the null hypothesis that the intercept is the same across all the regions and sites, and the random intercept model is valid and essential. At the same time, the likelihood ratio tests comparing the random slope model with the model that contains only random intercepts did not show a statistically significant difference for both the region or site levels (the *p*-values are close to 1 for all these tests). As a consequence, the consideration of random slopes was not necessary, so we limited our model only to the random intercepts.

The intra-class correlation, interpreted as a ratio of the variance of FCM levels due to different sites (or bioregions) to the total variance of FCM, determines the contribution of sites (or bioregions) to the total variance of FCM. That the intra-class correlation is equal to ∼0.26 for the site level, and ∼0.11 for the bioregion level means that the contribution of different sites to the variability of cortisol was significantly larger than that of different bioregions, indicating particularly strong clustering of the data with respect to the collection sites.

**Figure 3:**
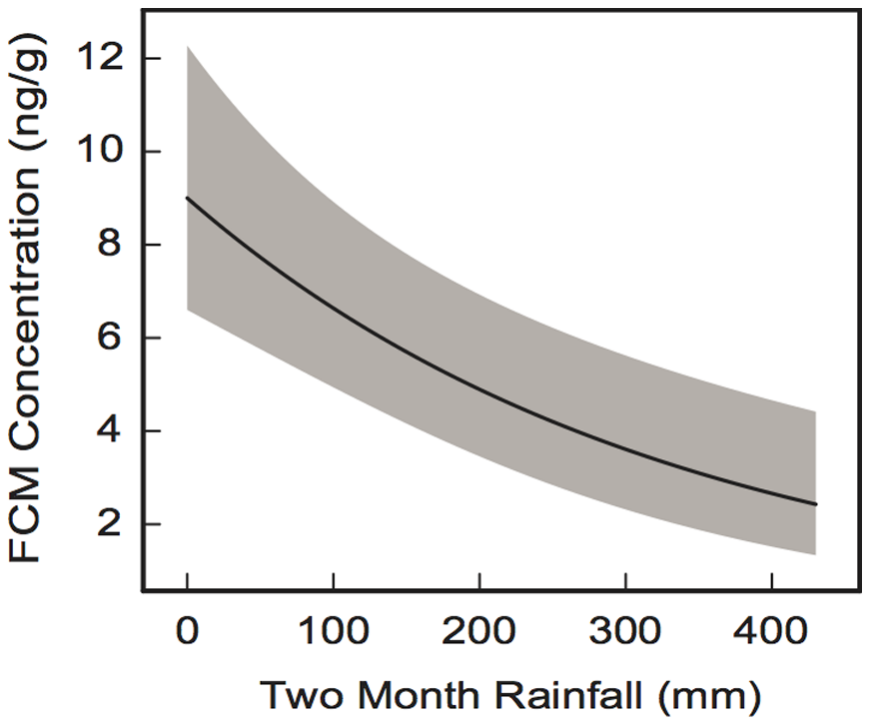
Faecal cortisol metabolite (FCM) concentration versus rainfall during the two months before the collection. The grey band shows the 95% prediction interval. The total rainfall for the previous 4 months (months 3 to 6) is assumed to equal its average value for that period of 310 mm.

The dependence of the average FCM level on rainfall for the two-month period prior to the collection, adjusted for rainfall during the following months 3 to 6, is significant and demonstrates a monotonic decrease of FCM levels with increasing two-month rainfall (Figure 3). For a two-month rainfall of 400 mm, with the total rainfall for the previous 4 months being equal to its average value of 310 mm, the average FCM level is ∼2.66 ng/g. If the two-month rainfall decreased from 400 mm to 50 mm, the FCM levels increased ∼3 times to ∼7.73 ng/g (Figure 3).

**Figure 4:**
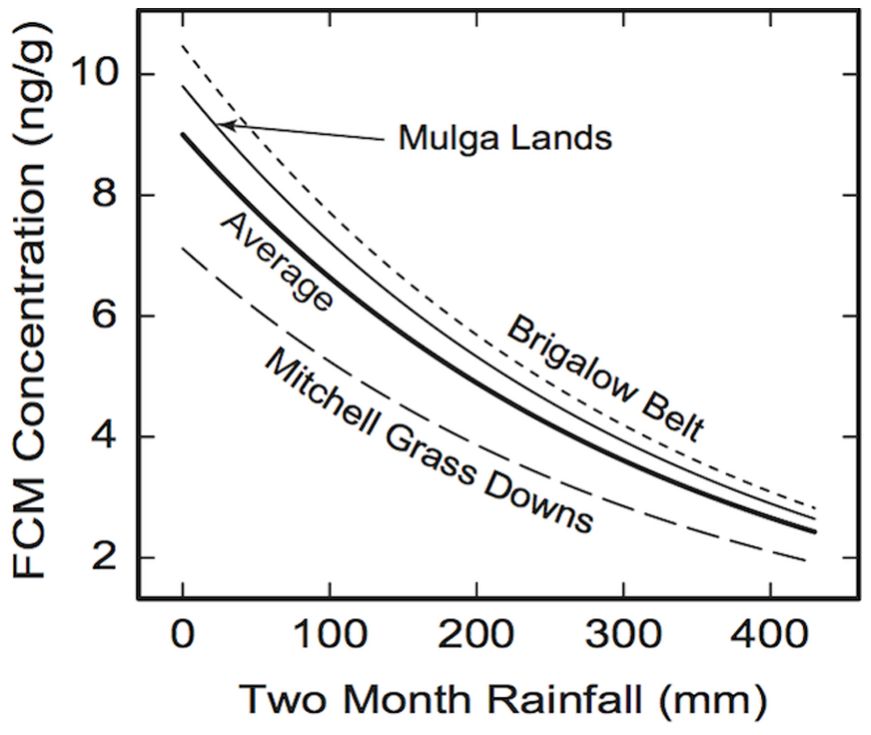
Regional variation in the relationship between faecal cortisol metabolite (FCM) concentration and rainfall during the two months before sample collection. The thick solid curve shows the cortisol levels averaged over all three bioregions combined (the same as the curve in Figure 3). The other three curves represent the average FCM levels for each of the bioregions: Brigalow Belt (small dashed curve), Mitchell Grass Downs (dashed curve), and Mulga Lands (thin solid curve). The total rainfall for the previous 4 months (months 3 to 6) is assumed to equal its average value of 310 mm.

The typical differences between the cortisol dependences for different bioregions are characterized by significantly lower levels of FCM in koalas at any level of rainfall (Figure 4). The model of the influence of rainfall on FCM concentration in koalas can account for ∼10% of the total FCM variability. The relatively small fraction of the total FCM variability, explained by the rainfall variables, does not mean that this effect is insignificant. As demonstrated, the results are statistically significant with low *p*-values. Further, the contribution of the rainfall variables is comparable with the variability of FCM by region (also ∼10%) and by site (∼14%). This means that the other ∼66% of FCM variability is due to other unexplained factors.

To further assess the fit of the models, 12 quantile-quantile (Q-Q) plots of the standardized residuals were considered for rainfall during the periods from 1 month to 12 months prior to sample collection. These plots further demonstrated the validity of the considered models (Figure S1).

## Discussion

Chronic stress can reduce resistance to disease and affect population performance (including impacting negatively on survival and reproduction) [35], [101]–[105], thus understanding the physiological responses of populations exposed to environmental changes is critical to conservation. We show that higher FCM levels in koala populations near the arid edge of their range in western Queensland were associated with lower rainfall levels, consistent with physiological stress when moisture levels are low. These results are of particular concern to the continued survival of western koala populations, especially when a number of studies have attributed population declines of free-living mammals to the effects of chronic stress [36], [83], [84]. Droughts and heatwaves have been responsible for population crashes and local extinctions in a number of koala populations [71], [86]–[88]. Predictions indicate that the intensity and frequency of droughts and heatwaves will increase and moisture availability will decrease [89]–[91]. Our findings show that western koalas, at the trailing edge of their geographic range, will cope poorly with changes in rainfall patterns resulting from climate change.

Arid and semi-arid landscapes are temporally and spatially variable, heterogeneous ecosystems where ecological processes are primarily driven by climate and nutrients [106]. Previous research within these arid and semi-arid landscapes has determined that the distribution, density, habitat preferences and home range sizes of koalas are affected either by water availability (including leaf moisture) and/or rainfall [75], [76], [86], [107], [108]. The findings from the current research have also demonstrated the impact of rainfall on koala populations by showing that low rainfall negatively impacts on FCM levels. In particular, rainfall from the previous two months had the strongest influence on FCM levels. Time lags can occur between rainfall events and the subsequent response by vegetation, with previous studies showing that total rainfall over the preceding two months has a strong effect on vegetation growth [96]–[98]. This indicates that the influence of rainfall on FCM levels in koalas is likely to be caused by the improved leaf moisture and nutrition in the form of new growth resulting from the rainfall. The delay of the new growth after a rainfall causes the consequential time lag of about two months between the rainfall and the maximum reduction in FCM levels in koalas. Foliar moisture supplies most of the koala’s water requirements. It has been proposed that in dry environments or during drought water, rather than leaf nutrients, influences tree selection by koalas [107], [109]. Therefore, rainfall two months prior is an essential determinant of leaf moisture content, and hence food quality, which in turn impacts on FCM concentrations of southwestern Queensland koalas. Results from this study are consistent with studies on African elephants, muriqui monkeys and spider monkeys [77], [79], [110]. These studies reported FCM concentrations significantly higher in the dry versus the wet season as food and/or water availability declined [77], [79], [110]. For example, FCM concentrations of free-ranging African elephants were significantly higher in the dry season as food, water availability and body condition declined and were inversely correlated with rainfall across seasons [79]. The authors also noted that elephant diets change from high-quality grass in the wet season to less nutritious and more sparsely distributed bark and leaves in the dry season [111], [112]. Spider monkeys in conserved forests also show seasonal variation in faecal cortisol concentrations, with higher concentrations measured in the dry season compared to the wet season, when there is a reduction in the number of fruiting trees and hence food availability [110]. The same study also found that mean cortisol concentrations of spider monkeys living in fragmented habitat remained high throughout the year, indicating that individuals within these habitats may suffer from long-term stress [110]. In addition, serum cortisol was significantly higher in camels in drought-affected areas than camels in other areas [113].

When comparing cortisol concentrations in relation to prior rainfall, FCM concentrations of koalas from the Mitchell Grass Downs were below the combined bioregion average, whereas FCM concentrations of koalas from the Mulga Lands and Brigalow Belt South were above the average. The dominant soil type of sites within the Mitchell Grass Downs (Vertosol) has high water holding capacity and very high soil fertility compared to the dominant soils of the Mulga Lands (Kandosol) and Brigalow Belth South (Sodosol, Chromosol and Vertosol) bioregions [114]–[116]. The difference in soil water holding capacity and fertility among the regions could explain why the Mitchell Grass Downs koalas had lower FCM concentrations, i.e. trees within this region are likely to have higher leaf moisture and forage quality as a result of the higher water holding capacity and fertility of the soil. A previous study within southwestern Queensland found that there was a significant relationship between leaf moisture and soil types, although once soil types were divided into groups based on water availability, the authors suggested the relationship could be ascribed to water availability in sites [117]. Furthermore, in this study, soil properties had no significant impact on the levels of FCM. Therefore, although soil water-holding capacity and/or fertility may explain the lower FCM concentrations of the Mitchell Grass Downs compared to the other two bioregions, rainfall two months prior is most likely the principal determinant of leaf moisture content and quality, regardless of soil type.

Significant effects of temperature on glucocorticoid concentrations have been shown for several mammalian species, with most studies reporting higher levels during harsher conditions in winter or during extreme heat [118], [119]. For example, a significant negative relationship was found between minimum ambient temperature and FCM levels of red deer [118]. In Marwari sheep, both hot ambient temperatures (maximum of 43–46°C) and drought conditions resulted in a significant increase in serum cortisol [119]. However, we found no impact of minimum and maximum ambient temperatures on FCM levels of koalas. Behavioural adjustment and physiological adaptations of koalas living near the arid edge of their range help mitigate the physiological stress of extreme temperatures. In central Queensland, the temperature in trees used by koalas for shelter in summer can be around 2°C cooler than the ambient temperature [71], and in Gunnedah, in north-western New South Wales, tree choice was found to be mediated by temperature [120]. Furthermore, the pelt of the koala has the highest recorded insulation (including a high resistance to wind) for a marsupial [121]. The dorsal surface is more densely furred and less reflective of solar radiation than the ventral surface [121]. Degabriele [121] suggested that different postural adjustments, particularly in relation to wind direction, allow for insulation capabilities, and that the field water influx and low metabolic rate of koalas are likely to be adaptations to their arboreal, low-energy folivorous lifestyle that extends over a wide latitudinal range. However, throughout the koala’s distribution, droughts and heatwaves have been responsible for population crashes and local extinctions [71], [86]–[88]. Although the metabolic and thermoregulatory adaptations of koalas help mitigate the effect of temperature on physiological stress, the studies to date identify that there is a threshold that, once crossed, cause population crashes. In this study, due to the relatively small number of samples collected at the lower and higher temperature extremes, this influence of temperature was not detected.

Variation in the physiological stress response, that influences the concentration of fecal GCMs, has been reported to reflect reproductive status or phase (i.e. oestrous, gestation, lactation) for a number of mammalian species [81], [122]–[124]. Glucocorticoids are also known to rise significantly near term in mammalian species because they trigger the cascade resulting in parturition [12], [125]. While these large changes in plasma cortisol occur in most eutherian mammals during gestation, the different endocrine control of parturition following the well known short gestation period in marsupials is not associated with any large changes in maternal plasma cortisol [126]. Consistently we did not detect differences between sexes or reproductive condition in this study. It is possible that during drought years, reproduction might have been suspended or reduced in southwestern Queensland koala populations, while as rainfall increases a normal pattern of reproduction might have been found. Indeed anecdotal evidence suggests such a scenario with only one mother and joey found during koala searches during the drought compared with numerous observations of mothers with joey’s post-flood. However, we found the opposite with higher FCM during low rainfall periods (consistent with moisture stress) compared with periods of increased rainfall when females might be in reproductive condition. Narayan *et al* [55] reported that FCM levels of captive koalas significantly differed by sex, reproductive condition (lactating versus non-lactating koalas) and handling groups; however, *post hoc* comparisons revealed that although FCM levels were significantly different between the sexes and handling groups, there was no significant differences between the reproductive conditions. Narayan *et al* [55] also reported no significant difference in FCM levels between wild and captive koalas, with *post hoc* comparisons further supporting the suggestion that within each environment (captive or wild) there was a significant effect of sex but not reproductive condition on the levels of FCM. Sex differences in either plasma free cortisol or fecal cortisol were not apparent in a study on captive koalas by Davies *et al* [54], however, these animals were not lactating and not known to be pregnant at the time of the study. Because of the large difference between eutherian and metatherian reproductive endocrine control, the large changes in maternal plasma cortisol that occur in most eutherians during pregnancy do not occur during the short gestation in marsupials and fetal cortisol is more important for initiating parturition and enabling development and maturation of organs (in particular the lungs) in preparation for parturition [126]. Since this study measures a downstream excretory product of hepatic clearance of plasma free cortisol, not total cortisol, sex differences are likely to be small. This adds further support to the suggestion that rainfall, and the subsequent leaf moisture and/or leaf nutritional content, is the principal determinant of FCM levels of southwestern koala populations. However, future studies should address possible differences between sexes and reproductive status and to do this thoroughly, steroid partitioning in the blood plasma must be assessed enabling calculation of free cortisol.

Sustained high levels of glucocorticoids from chronic stress (e.g. from long-term climate change) can be deleterious to the physiological health of an animal and lead to greater susceptibility to disease, as well as reduced fecundity and survivorship [12], [13], [20], [34], [35]. While the deleterious effects of chronic stress are well conceptualized from biomedical evidence, there is some doubt whether or not these deleterious effects, especially relating to pathology, occur in nature [41], [127]. Boonstra [41] argues that both demographic (the indirect effects on reproduction) and hormonal evidence indicate that although some wild populations may be chronically stressed by predators, there is no evidence of pathology or an HPA axis with an inability to respond. Boonstra [41] suggests that chronic stress is not the inevitable result of a persistent, severe stressor, but is part of the normal experience of many animals in nature and, although there are fitness costs, it is an evolved adaptive response to particular ecological and habitat pressures. While this may be the case for predation stressors, there is no evidence in wild populations whether this adaptive response can be applied to other severe chronic stressors of a different nature, such as climate change or habitat loss and fragmentation. For species that have populations threatened by disease, chronic stress acting via well known effects on the immune system, can be expected to increase susceptibility to disease and threaten the long-term viability of populations. Koalas are subject to a range of diseases, which can lead either to death or infertility [128], [129]. Infectious diseases in koalas are recognised to threaten the long-term viability of populations in Queensland and New South Wales [129]. In northwestern New South Wales, the prevalence of the disease chlamydiosis was found to increase dramatically following intense stress from extreme hot weather (intense heatwaves) during a drought [87]. Following a review of studies of chlamydial disease and population decline, Lunney *et al.* [87] also linked chlamydiosis to koala populations under stress from habitat loss and fragmentation. Furthermore, Gordon [86] reported that on dry stretches of creek in southwestern Queensland koalas showed poorer health (poor condition, anemia, high tick loads) and suffered much higher mortality, i.e. population crashes, during drought compared to sites where trees were not affected by the drought (mainly on large permanent waterholes). We found low rainfall resulted in high cortisol levels, demonstrating that droughts have the potential to impact on the physiological stress of individual koalas as a result of reduced food and water availability. It is increasingly likely exposure to these environmental stressors will increase their susceptibility to disease. However, although there is anecdotal evidence reporting that stress and disease prevalence in various koala populations throughout its distribution are related, the link between chronic stress, disease and ensuing demographic impact needs detailed examination before any causal links can relate chronic stress to pathology. Our results provide new insights into the response of koalas to stressors, such as drought and heatwaves, and highlight the conservation concerns for the continued survival of western koala populations in light of predicted climate change.

### Implications for Conservation

Drought and heatwaves have the potential to impact on physiological stress in individual koalas as these extreme climatic conditions can affect the quality of nutrients and moisture available in the diet of entire populations [130], [131]. Observed FCM concentration patterns in relation to rainfall levels strongly suggest that physiological stress in western koalas is being adversely affected by limited access to food and water. These western Queensland koala populations are at the semi-arid edge of their distribution, where drought and heatwaves have resulted in population declines [86], [88], which confirms that koalas in this semi-arid region are near their physiological tolerance limits. Climate change predictions for this region indicate that the intensity and frequency of droughts and heatwaves will increase, evapotranspiration will increase, and that foliar moisture will decrease [89]–[91]. This highlights conservation and management concerns for the continued survival of koala populations of western Queensland.

Investigating FCM levels at the trailing edge of a widely distributed species boundary, and how these levels vary with climatic factors, allows us to non-invasively identify changes likely to cause stress. It also allows us to identify the habitats that are least susceptible to climate change as priority sites for protection, as well as to facilitate management decisions to prevent further contractions in a species’ distribution, and monitor the efficacy of management strategies. In southwestern Queensland, riparian habitat is the primary resource used by koalas [88], [95], [108], based on evidence of populations declining and contracting to riparian habitats during drought and heatwaves [86], [88]. It follows that since the moisture content of leaves in trees within riparian habitats would be higher, it makes them higher quality habitat for koalas. Considering that low rainfall, and hence low leaf moisture, resulted in higher FCM levels, it places even more importance on maintaining and improving the quality of riparian habitats for continued survival of these western koala populations, particularly during extreme weather. Historical land management practices have diminished riparian habitats from the silting of previously-permanent water-holes [86]. For the long-term conservation of western koala populations, fencing of riparian habitats to keep out feral animals and domestic stock should be considered. This will help to reduce the silting of waterholes and allow the recruitment of food trees. However, reduced grazing could also increase weed species, which could negatively impact on koalas and other native wildlife. This points to the need for close monitoring and control of weed infestations. Previous studies have also found water availability and rainfall to be the main factors defining habitat preference of koalas in arid and semi-arid regions [75], [107]. Maintaining access to freestanding water, such as farm dams, and planting favoured tree species, particularly those that tend to have higher leaf moisture content, such as *E. camadulensis* and *E. coolabah* [117]), close to dams would also increase the availability of high quality trees, as these trees are likely to have higher leaf moisture levels. Therefore, to ensure the continued survival of western Queensland koala populations in extreme weather, the most important actions for koala conservation within these semi-arid landscapes are the maintenance of the quality and quantity of riparian habitats, expanding the availability and accessibility of freestanding water, such as around farm dams, and providing specific trees as food resources.

## Conclusion

Ecological studies are increasingly using the release of glucocorticoids as a measure of the stress response in animals. Using non-invasive methods to measure the glucocorticoid metabolites in faeces, we found that koalas had higher FCM levels during low rainfall periods. An analysis of mechanisms for the impact of rainfall on FCM levels in koalas is beyond the scope of this paper, but one mechanism could be through the new growth and higher foliar moisture content of eucalypt leaves during periods of rainfall. This would provide koalas with an abundance of food and water causing reductions of their cortisol levels. These results provide new insights into the effect of droughts and heat waves on physiological stress levels and highlight conservation concerns for the continued survival of western Queensland koala populations in the face of predicted climate change. Our results demonstrate the importance of integrating a physiological dimension into ecological studies to ensure a more comprehensive understanding of species’ responses to habitat loss, particularly riparian habitat, and the increasing threat of relentless climate change.

## Supporting Information

Figure S1 **Q-Q plots of the standardized residuals for the models for (a) rainfall 1 month prior to sample collection, (b) rainfall 2 months prior to sample collection, (c) rainfall 3 months prior to sample collection, (d) rainfall 4 months prior to sample collection, (e) rainfall 5 months prior to sample collection, (f) rainfall 6 months prior to sample collection, (g) rainfall 7 months prior to sample collection, (h) rainfall 8 months prior to sample collection, (i) rainfall 9 months prior to sample collection, (j) rainfall 10 months prior to sample collection, (k) rainfall 11 months prior to sample collection, and (l) rainfall 12 months prior to sample collection.**
(DOCX)
