# Supplementary figures and images for: Physiological Stress in Koala Populations near the Arid Edge of Their Distribution
Source: PLoS One. 2013 Nov 12;8(11):e79136. doi: 10.1371/journal.pone.0079136 (PMC3827162; doi:10.1371/journal.pone.0079136)

(a)
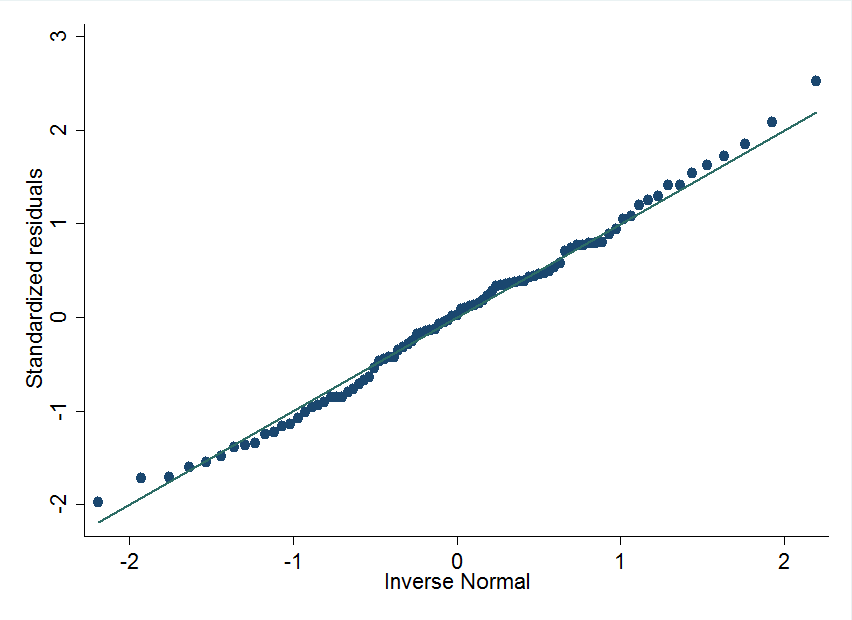


(b)
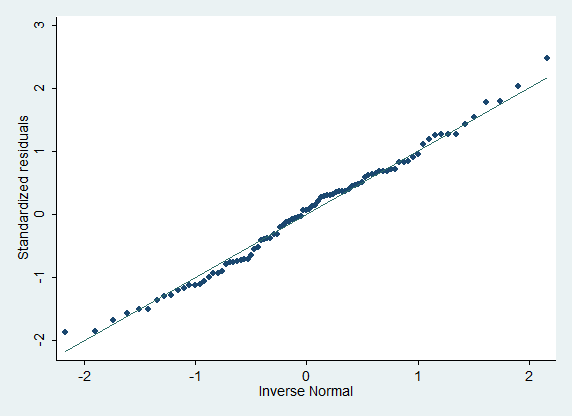


(c)
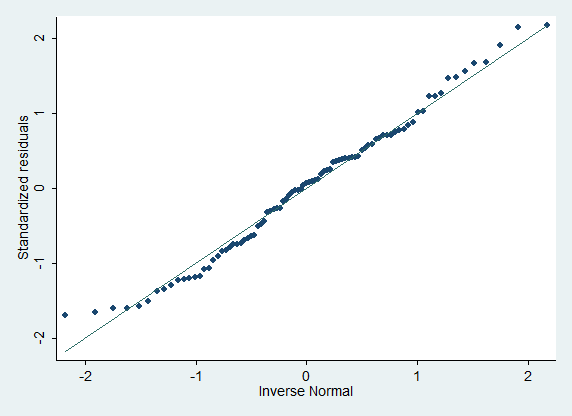


(d)
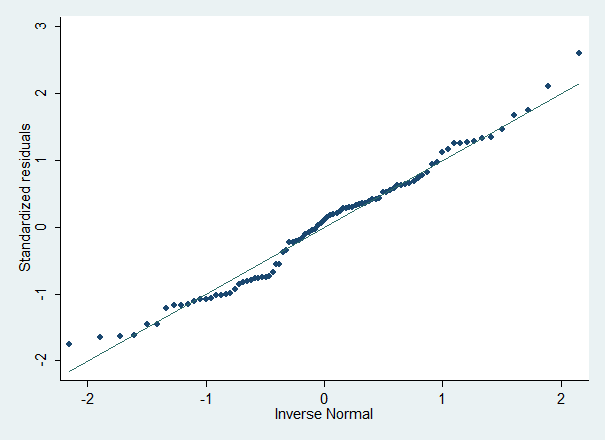


(e)
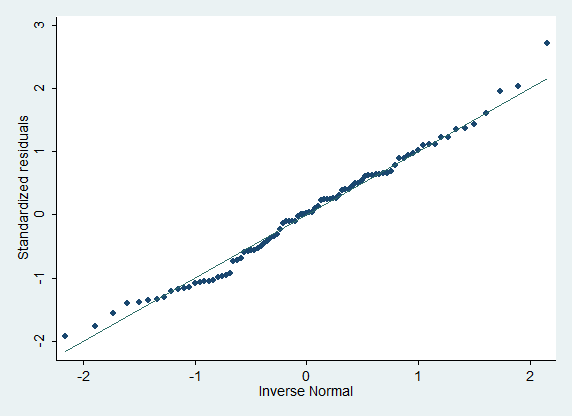


(f)
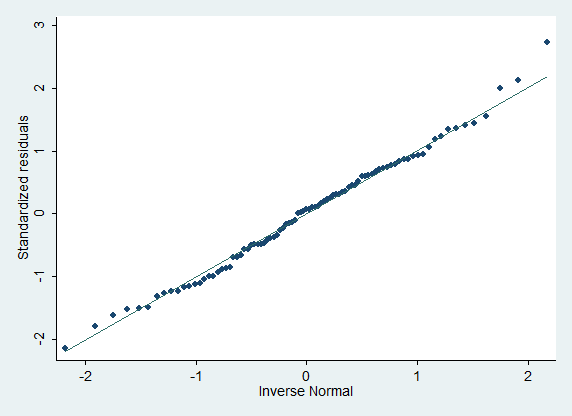


(g)
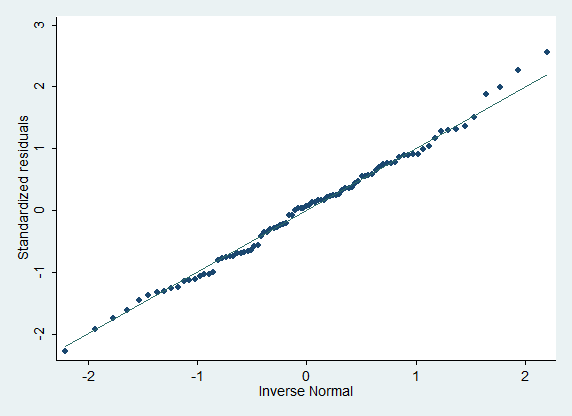


(h)
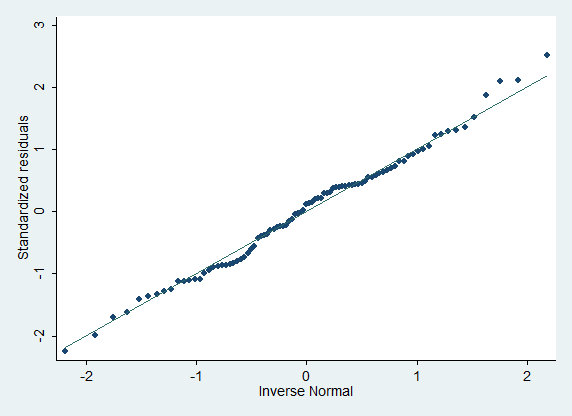


(i)
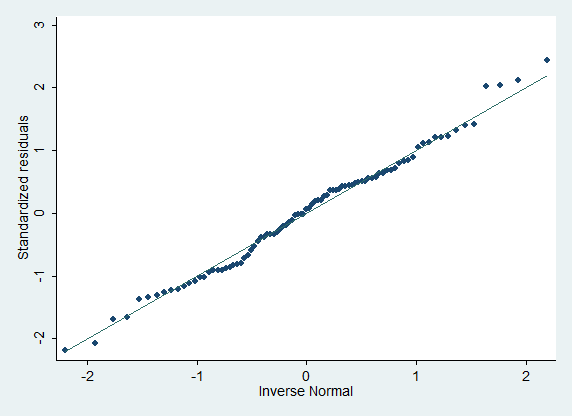


(j)
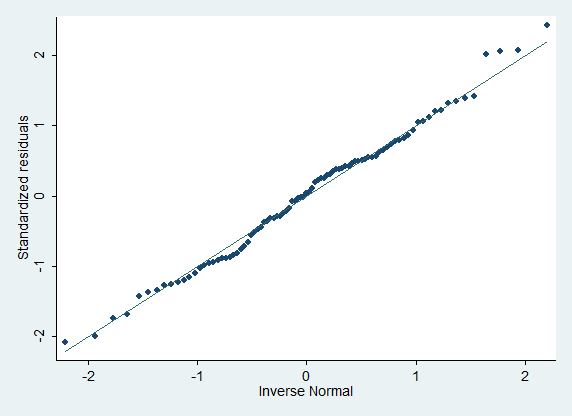


(k)
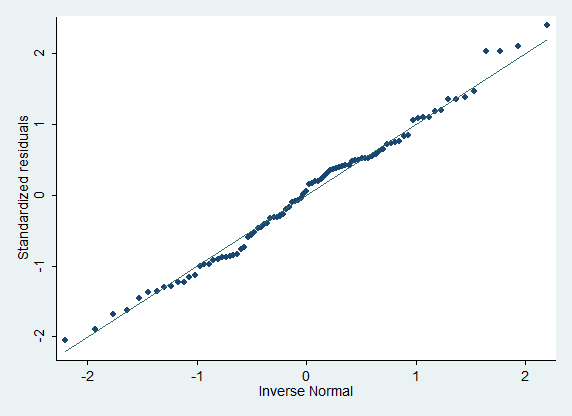


(l)
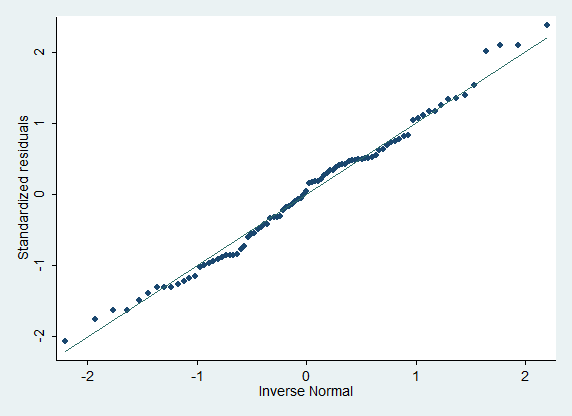

Supplement: Figure S1 — Q-Q plots of the standardized residuals for the models for (a) rainfall 1 month prior to sample collection, (b) rainfall 2 months prior to sample collection, (c) rainfall 3 months prior to sample collection, (d) rainfall 4 months prior to sample collection, (e) rainfall 5 months prior to sample collection, (f) rainfall 6 months prior to sample collection, (g) rainfall 7 months prior to sample collection, (h) rainfall 8 months prior to sample collection, (i) rainfall 9 months prior to sample collection, (j) rainfall 10 months prior to sample collection, (k) rainfall 11 months prior to sample collection, and (l) rainfall 12 months prior to sample collection. (DOCX) [file pone.0079136.s001.docx]
